# Potential of gut microbiota metabolites in treating COPD: network pharmacology and Mendelian randomization approaches

**DOI:** 10.3389/fmicb.2024.1416651

**Published:** 2024-11-25

**Authors:** Zhenghua Cao, Shengkun Zhao, Tong Wu, Feng Sun, Shaodan Hu, Li Shi

**Affiliations:** ^1^Graduate School, Changchun University of Chinese Medicine, Changchun, Jilin, China; ^2^Geriatric Department, Suzhou Hospital of Integrated Traditional Chinese and Western Medicine, Suzhou, China; ^3^Respiratory Disease Department, Affiliated Hospital of Changchun University of Chinese Medicine, Changchun, Jilin, China

**Keywords:** network pharmacology, MR, COPD, biological network, *Phenylacetylglutamine*

## Abstract

**Objective:**

The gut microbiota and its metabolites exert a significant influence on COPD, yet the underlying mechanisms remain elusive. We aim to holistically evaluate the role and mechanisms of the gut microbiota and its metabolites in COPD through network pharmacology and Mendelian randomization approaches.

**Methods:**

Employing network pharmacology, we identified the gut microbiota and its metabolites’ impact on COPD-related targets, elucidating the complex network mechanisms involving the gut microbiota, its metabolites, targets, and signaling pathways in relation to COPD. Further, promising gut microbiota metabolites and microbiota were pinpointed, with their causal relationships inferred through Mendelian randomization.

**Results:**

A complex biological network was constructed, comprising 39 gut microbiota, 20 signaling pathways, 19 targets, and 23 metabolites associated with COPD. *Phenylacetylglutamine* emerged as a potentially promising metabolite for COPD treatment, with Mendelian randomization analysis revealing a causal relationship with COPD.

**Conclusion:**

This study illuminates the intricate associations between the gut microbiota, its metabolites, and COPD. *Phenylacetylglutamine* may represent a novel avenue for COPD treatment. These findings could aid in identifying individuals at high risk for COPD, offering insights into early prevention and treatment strategies.

## 1 Introduction

Chronic Obstructive Pulmonary Disease (COPD) is a heterogeneous disorder primarily characterized by airway pathologies (bronchitis, bronchiolitis) and/or alveolar abnormalities (emphysema) leading to chronic respiratory symptoms (dyspnea, cough, sputum production) and progressive, persistent airflow limitation [Global Initiative for Chronic Obstructive Lung Disease (GOLD), 2024]. COPD has ascended to become the third leading cause of death globally (WHO, 2024), representing a significant public health challenge. Studies indicate that COPD has a prolonged latency period (Divo et al., 2023), with both its prevalence and mortality rates on an upward trajectory annually (Adeloye et al., 2022). Research targeting middle-aged individuals reveals that the prevalence of COPD in those over 30 years of age approximates 11.7% (Adeloye et al., 2015). Furthermore, studies focusing on the prevalence of COPD in China demonstrate that among individuals aged 40 and above, the prevalence rate soars to 13.7%, with the total number of affected individuals nearing 100 million (Wang et al., 2018).

The gut microbiome, often referred to as the body’s “second genome,” stands at the core of human health (Dogra et al., 2020) and may serve as a crucial intermediary between disease and the evolution of the human genome (Quan et al., 2023). A dynamic equilibrium exists between a healthy gut microbiome and the host, with the microbiome being associated with a multitude of diseases (Balint and Brito, 2024) and offering insights into disease at various functional levels (Muller et al., 2024). The composition and variations of the gut microbiome and its metabolites can influence the function of distant lungs (Dokoshi et al., 2024). There is a correlation between the gut microbiome and chronic pulmonary diseases through the gut-lung axis (Sun et al., 2024a), making it an indispensable participant in respiratory immunity and inflammation (Perdijk et al., 2024), though the mechanisms remain unclear. Dysbiosis of the gut microbiome can affect the development of COPD (Li N. et al., 2021), and there is an association between gut microbiome imbalance and COPD (Liu P. et al., 2024). Studies indicate that the gut microbiome is involved in the mechanisms of COPD and could potentially be a target for COPD-specific therapies (Budden et al., 2024). There is a correlation between the gut microbiome and airway inflammation (Frayman et al., 2024), and the gut microbiome may play a role in defending against respiratory viruses in the lungs, possibly by influencing alveolar macrophages to phagocytize influenza viruses, thus better protecting the lungs (Wang et al., 2024b). Examination of the gut microbiome in COPD patients and healthy individuals revealed 146 differences, with *Lachnospiraceae* potentially linked to reduced lung function (Bowerman et al., 2020). Research has found that the gut bacterium *Parabacteroides goldsteinii* may improve COPD by affecting mitochondrial activity, amino acid metabolism, and reducing lung inflammation (Lai et al., 2022b). Smoking leads to an increase in *Streptococcus* in the gut and reduces the diversity of the gut microbiome (Shanahan et al., 2018). Patients with severe COPD tend to have an abundance of *Fusobacterium* and *Aerococcus*, and the gut microbiome can influence COPD by shaping the immune system (Chiu et al., 2021). Gut microbiome metabolites, such as short-chain fatty acids, have anti-inflammatory activities (Dang and Marsland, 2019), are fundamental mediators in the gut-lung axis, and participate in various lung diseases (Ashique et al., 2022), regulate immune homeostasis (Goncalves et al., 2018), and can improve lung function and suppress inflammation (Wang J. et al., 2023). *Butyrate* can inhibit airway inflammation in COPD patients (Jiang et al., 2024), and *arginine* can modulate inflammation (Fritz, 2013), which is associated with lung function (Maarsingh et al., 2008; Halper-Stromberg et al., 2019). Gut microbiome metabolites can reduce lung inflammation induced by cigarette smoke (Nascimento et al., 2023), and *acetate* and *propionate* can decrease alveolar damage and inflammation (Lee et al., 2023). Metabolites of the gut microbiome could act as a pivotal link between the microbiome and the gut-lung axis, harboring the potential to treat respiratory diseases by controlling lung inflammation from a dysbiosis perspective (Nascimento et al., 2023). By modulating the gut-lung axis and macrophage polarization, it shapes the immune response in the lungs and is related to the pathogenesis of respiratory diseases (Chen et al., 2023). The gut microbiome, its metabolites, target actions, and diseases constitute a complex biological network, hence focusing on specific gut microbiome metabolites could aid in understanding the mechanisms of diseases.

In this study, the methodology of network pharmacology is employed to explore the relationships between the gut microbiome, its metabolites, targets, signaling pathways, and COPD from a holistic perspective. The aim is to identify the core gut microbiome, its metabolites, targets, and key signaling pathways that influence COPD. Furthermore, by utilizing the Mendelian randomization approach, the study delves into the causal relationships between the core gut microbiome, its metabolites, and targets with COPD, thereby dissecting the causality between these elements and COPD from an overarching standpoint.

## 2 Methods

### 2.1 Study design

**Figure 1:**
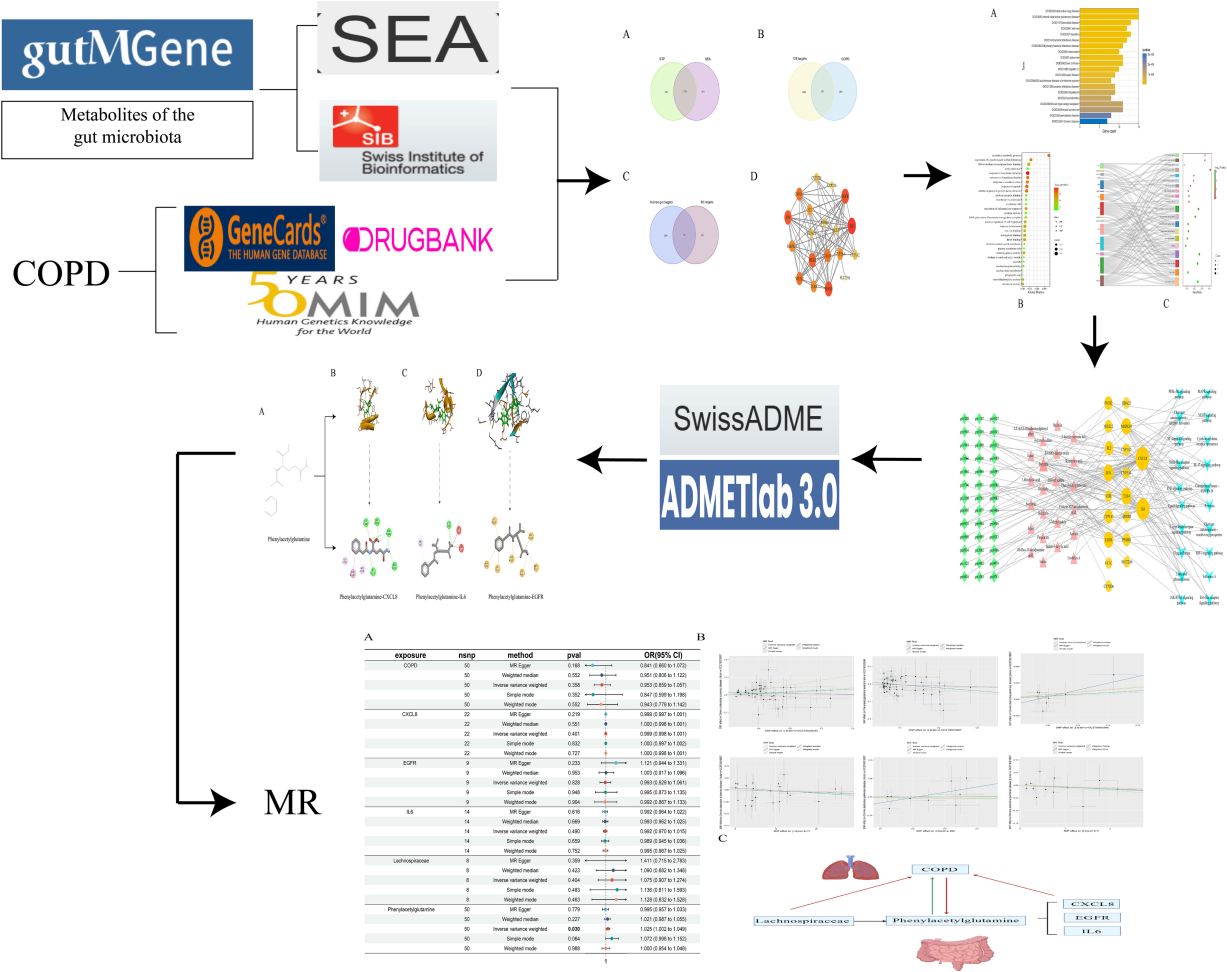
Research ideas.

Grounded in the principles of network pharmacology and Mendelian randomization, this study embarks on an investigation from the perspective of the gut-lung axis into the pivotal substances and potential mechanisms of gut microbiome metabolites in COPD. Employing the method of Mendelian randomization, it further probes into the causal relationships between relevant gut microbiome metabolites, upstream gut microbiota, and downstream related genes with COPD. By examining the impact of the gut microbiome on COPD from a comprehensive viewpoint, this research lays the theoretical groundwork for subsequent studies focused on treating lung conditions through interventions targeting the gut (Figure 1).

### 2.2 Target acquisition of gut microbiota metabolites and COPD

In the quest to identify the metabolic products of the gut microbiome and their targets in relation to COPD, we harnessed the gutMGene v1.0 database^1^ to search for metabolites and targets of the gut microbiome (Cheng et al., 2022). Canonical SMILES of these metabolites were sought through PubChem^2^ and subsequently inputted into the Similarity Ensemble Approach (SEA^3^) (Keiser et al., 2007) and Swiss Target Prediction (STP^4^) (Daina et al., 2019) platforms to predict all related targets of gut microbiome metabolites. An intersection of results from both platforms yielded the relevant targets of gut microbiome metabolites.

Utilizing databases such as GeneCards,^5^ OMIM,^6^ and DRUGBANK^7^ with “COPD” as the keyword, we filtered for disease targets related to COPD. The GeneCards database was sifted through based on the median value of the “relevance score.” After integrating the three aforementioned databases and eliminating duplicate targets, a COPD target database was constructed.

By intersecting the related targets of gut microbiome metabolites with COPD targets, we identified significant targets of gut microbiome metabolites acting on COPD. Further intersection of these significant targets with targets from the gutMGene database elucidated the key targets through which gut microbiome metabolites affect COPD.

### 2.3 Construction and enrichment analysis of PPI networks

To illuminate the potential signaling pathways through which gut microbiome metabolites influence COPD, the initial step involves uploading the identified key targets to the String database.^8^ By setting specific parameters, a Protein–Protein Interaction (PPI) network is established. Following this, the Cytoscape 3.9.1 software is utilized to create a network diagram of these key targets, with the Degree values serving as a pivotal criterion for the construction. Further analytical depth is achieved through the enrichment analysis of these key targets using Gene Ontology (GO), Kyoto Encyclopedia of Genes and Genomes (KEGG), and Disease Ontology (DO). This step not only facilitates the visualization of these analyses but also aids in uncovering the underlying signaling pathways by which gut microbiome metabolites may affect COPD. This comprehensive approach provides a clearer understanding of the complex interactions between gut microbiome metabolites and COPD, highlighting the intricate biological processes involved.

### 2.4 The network diagram of gut microbiota-metabolites-target-signaling pathway was constructed

Utilizing the Cytoscape 3.9.1 software, we construct a network diagram that intricately maps the relationships between gut microbiota, metabolites, targets, and signaling pathways. Through the application of network analysis plugins, we meticulously analyze the interconnections among gut microbiota, metabolites, targets, and signaling pathways. This rigorous analysis enables us to pinpoint the most critical components within this network, including key gut microbiota, metabolites, targets, and signaling pathways, thereby enhancing our understanding of their roles and interactions in the biological system under study.

### 2.5 Evaluation of drug-likeness properties and toxicological evaluation by ADMETlab

Employing computational tools, we simulate the physicochemical properties of relevant metabolites using SwissADME^9^ to assess their drug-likeness (Daina et al., 2017), thereby determining their potential efficacy against COPD. Subsequently, these metabolites, characterized by their physicochemical properties, are further evaluated using ADMETlab 3.0.^10^ This evaluation encompasses a comprehensive toxicological assessment across eight dimensions: hERG blockers, Human hepatotoxicity (H-HT), Respiratory toxicity, Rat oral acute toxicity, Carcinogenicity, Drug-induced liver injury (DILI), Skin sensitization, and LD50 of acute toxicity. Through this meticulous process, we aim to identify the most suitable gut microbiome metabolites for potential therapeutic applications.

### 2.6 Molecular docking validation of metabolites of gut microbiota

The gut microbiome metabolites, having undergone rigorous drug-likeness evaluation and toxicological assessment, will be subjected to molecular docking with key targets to validate their interactions. The outcomes of these validations will be meticulously visualized, offering a clear depiction of the potential efficacy and interaction dynamics between the metabolites and the targets.

### 2.7 Mendelian randomization analysis of gut microbiota metabolites, gut microbiota, and related genes and COPD

The selected gut microbiome metabolites, along with their upstream gut microbiota and downstream relevant genes, will be subjected to Mendelian randomization to ascertain their causal relationship with COPD. The genetic information pertaining to the gut microbiome metabolites, their upstream microbiota, and downstream genes in relation to COPD is sourced from the GWAS database.^11^ To evaluate the causality, we employ five methods: Inverse Variance Weighted (IVW), MR-Egger, Weighted Median, Simple Mode, and Weighted Mode methods, with IVW serving as the primary method (Pierce and Burgess, 2013; Davies et al., 2018). A *p*-value of less than 0.05 indicates a causal relationship (Sanderson, 2021), while the other four methods serve as supplementary approaches (Burgess and Thompson, 2017). To assess the robustness of our results, leave-one-out sensitivity analysis is conducted, further complemented by tests for pleiotropy and heterogeneity, with a *p*-value greater than 0.05 indicating the absence of both pleiotropy and heterogeneity (Cai et al., 2022; Shi et al., 2022). To ensure the credibility of the results, we have validated the outcomes of the MR analysis using the Bayesian Weighted Mendelian Randomization (BWMR) method (Zhao et al., 2020; Grant and Burgess, 2024), to ascertain the accuracy of the causal relationship between the two variables. All analyses are performed using the R programming language (version 4.3.2).

## 3 Results

### 3.1 Gut microbiota metabolites act on key targets in COPD

**Figure 2:**
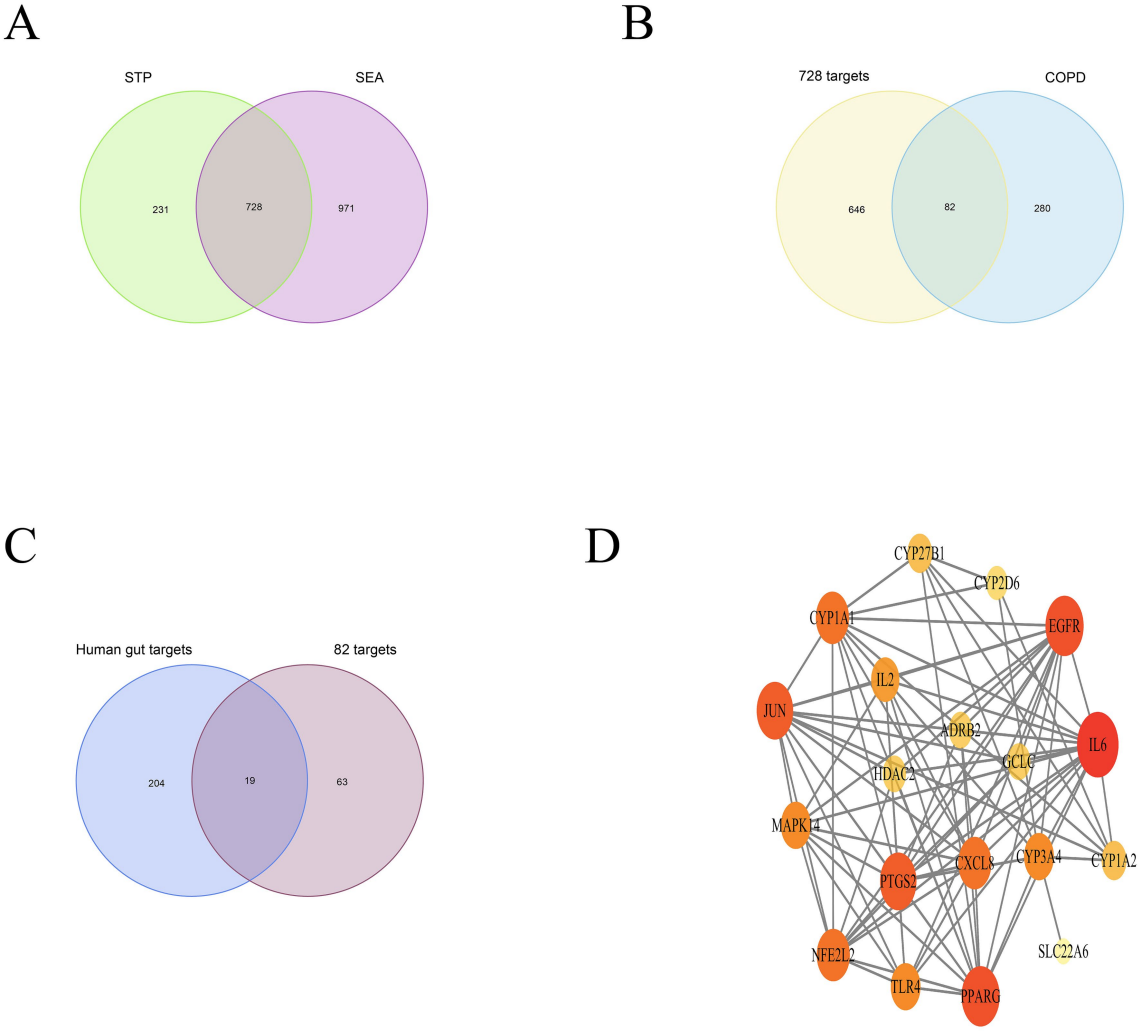
depicts the intersection between SEA and STP **(A)**; The overlap of gut microbiome metabolites with COPD **(B)**; Key targets **(C)**; PPI network **(D)**.

The gutMGene database version 1.0 encompasses 333 types of gut microbiota, 208 gut microbiome metabolites, and 223 targets. Through the application of SEA, a total of 1701 targets for gut microbiome metabolites were predicted, while STP forecasted 959 targets. An intersection of these predictions yielded 728 relevant targets for gut microbiome metabolites (Figure 2A). By filtering and eliminating duplicates from databases such as Genecards, OMIM, and DRUGBANK, we identified 362 targets related to COPD. An intersection of these with the previously mentioned 728 targets revealed 82 significant targets for gut microbiome metabolites that influence COPD (Figure 2B). Further intersection with the 223 targets listed in the gutMGene database pinpointed 19 critical targets through which gut microbiome metabolites affect COPD (Figure 2C).

### 3.2 PPI and enrichment analysis

**Figure 3:**
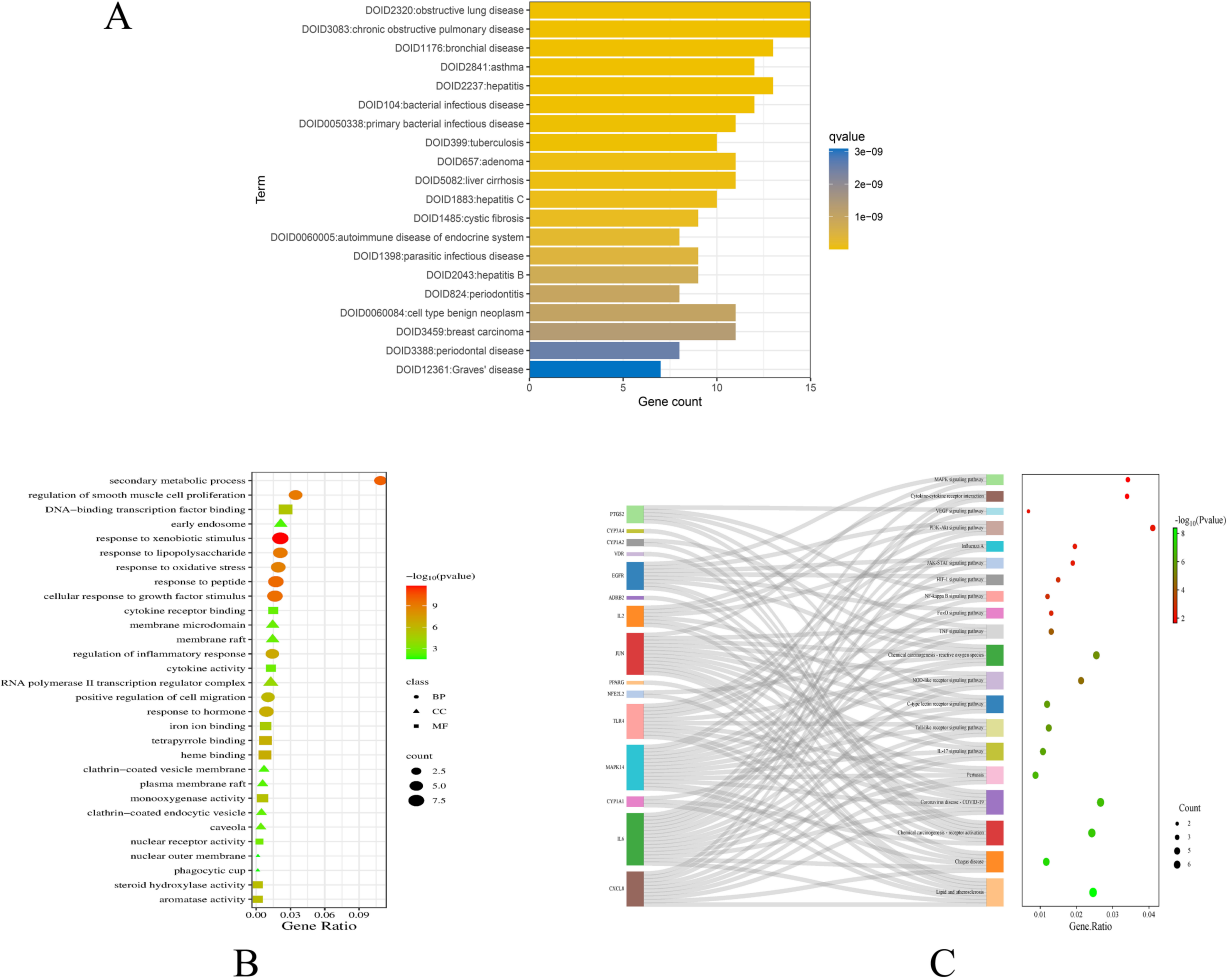
DO enrichment analysis bar chart **(A)**; GO enrichment bubble map **(B)**; Bubble map of KEGG enrichment pathways **(C)**.

The PPI network comprises 19 nodes and 85 edges, where the size of each node is determined by its degree value. IL6, EGFR, PPARG, PTGS2, JUN, and CXCL8 emerge as pivotal hubs within this PPI network (Figure 2D). To further assess the potential role of gut microbiome metabolites in the treatment of COPD, we conducted enrichment analysis on the 19 key targets of gut microbiome metabolites affecting COPD. The DO enrichment analysis primarily highlighted respiratory system diseases such as obstructive lung disease, chronic obstructive pulmonary disease, bronchial disease, and asthma, further affirming the gut microbiome’s role in modulating immunity and inflammation through the gut-lung axis in relation to chronic lung diseases (Figure 3A). This suggests that gut microbiome metabolites may serve as a communicative foundation between these systems (Wang X. et al., 2023).

GO enrichment analysis revealed significant involvement in processes such as phagocytic cup, nuclear outer membrane, membrane raft, and early endosome, participating in functions like iron ion binding, cytokine receptor binding, cytokine activity, and nuclear receptor activity. These processes are implicated in responses to lipopolysaccharide, regulation of smooth muscle cell proliferation, response to oxidative stress, regulation of inflammatory response, and response to hormone (Figure 3B). KEGG pathway enrichment analysis focused on Lipid and atherosclerosis, IL-17 signaling pathway, Toll-like receptor signaling pathway, TNF signaling pathway, and PI3K-Akt signaling pathway, with the analysis also highlighting key targets within these enriched pathways (Figure 3C).

### 3.3 Construction of network map of gut microbiota-metabolites-target-signaling pathway and screening of key components

**Figure 4:**
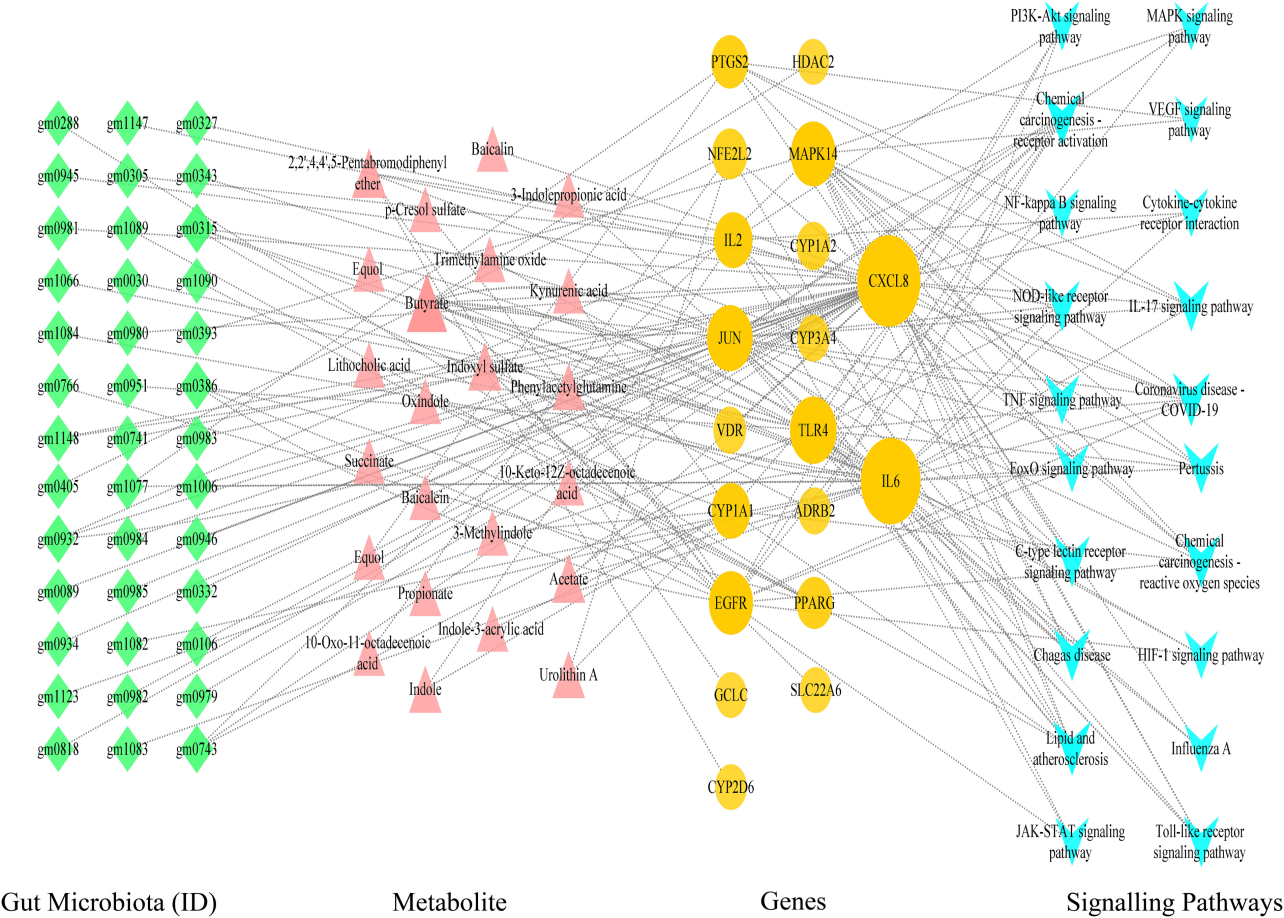
The network of the gut microbiome-metabolites-targets-signaling pathways.

Utilizing Cytoscape 3.9.1 software, we proceeded with the network analysis of gut microbiota-metabolite-target-signal pathways, encompassing 101 nodes (comprising 39 gut microbiota, 20 signal pathways, 19 targets, and 23 metabolites) and 182 edges. The network analysis unveiled that the foremost signal pathways include Lipid and atherosclerosis, Coronavirus disease-COVID-19, and the IL-17 signaling pathway. Predominant metabolites identified were *Butyrate* and *Acetate*, while leading gut microbiota comprised *Faecalibacterium prausnitzii* and *Lactobacillus rhamnosus*. Central targets highlighted were CXCL8, IL6, TLR4, JUN, MAPK14, and EGFR. For instance, the IL-17 signaling pathway was linked to 10 metabolites and 5 targets (IL6, JUN, CXCL8, PTGS2, and MAPK14), illustrating the intricate interplay between these components within the network (Figure 4).

### 3.4 Drug similarity and toxicity of associated metabolites in computer simulations

**Figure 5:**
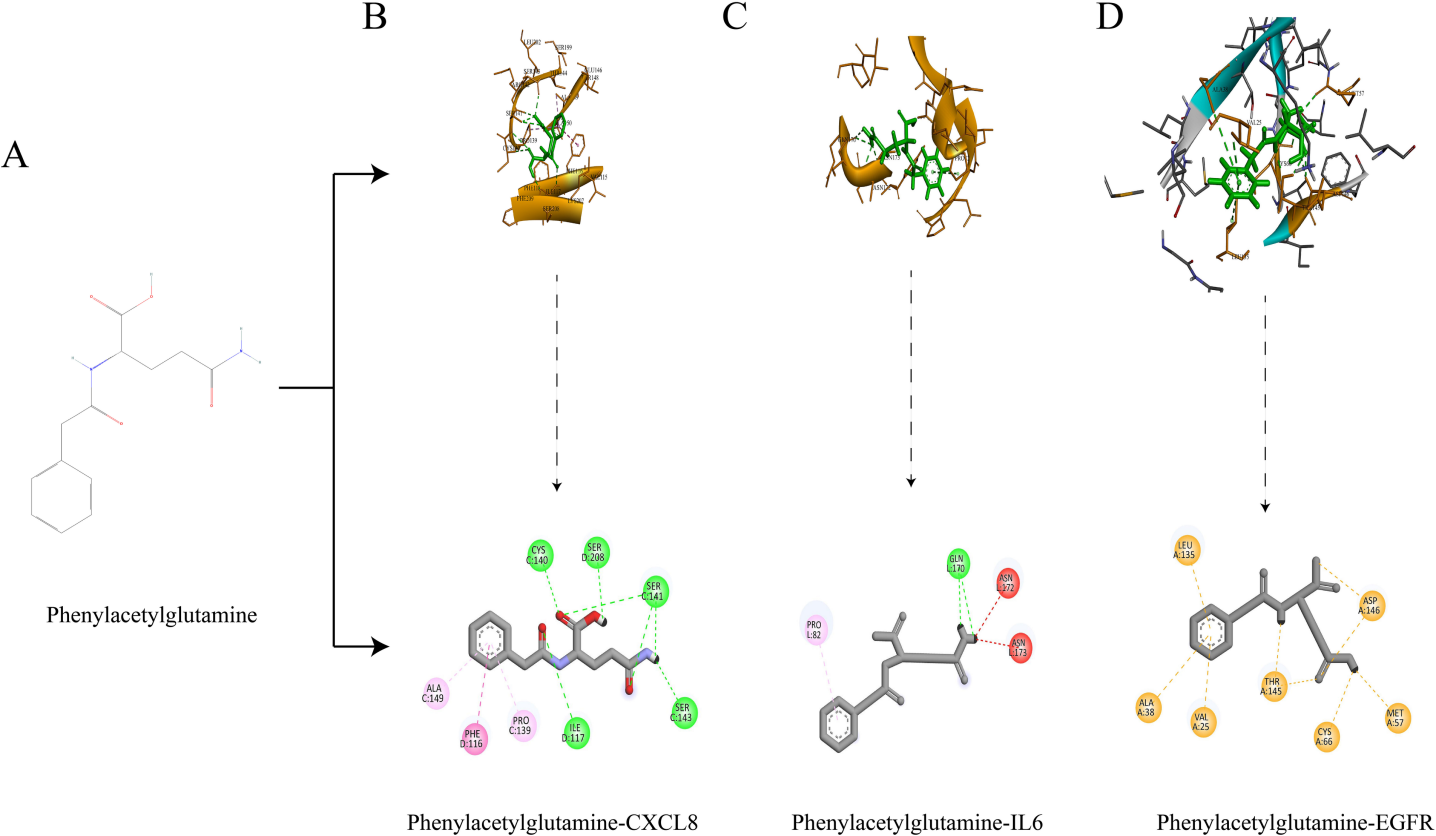
Structural formula of Phenylacetylglutamine **(A)**; Molecular docking of Phenylacetylglutamine with CXCL8 **(B)**; Molecular docking of Phenylacetylglutamine with IL6 **(C)**; Molecular docking of Phenylacetylglutamine with EGFR **(D)**.

In our computational analysis of the ADME parameters for 23 metabolites, we discovered that 20 of these metabolites exhibit drug-like properties. However, *Baicalin*, *Lithocholic acid*, and *2,2′,4,4′,5-Pentabromodiphenyl ether* were found to contravene Lipinski’s rule of five, leading us to conclude that 20 metabolites may possess potential in influencing COPD (Table 1). It is crucial to note that the physicochemical properties of metabolites do not represent safety indicators; toxicological evaluation plays a vital role in assessing safety. Therefore, we conducted a toxicological property assessment of the selected 20 metabolites using ADMETlab 3.0. Through this evaluation, *Phenylacetylglutamine* was identified as a promising metabolite (Figure 5A), potentially offering significant developmental value (Table 2).

**Table 1:** The physicochemical properties of the metabolites from gut microbiota.

NO.	Metabolites	Lipinski rule				Lipinski’s violations ≤1	Bioavailability score > 0.1	TPSA < 140 Å^2^
		MW ≤ 500	HBA ≤ 10	HBD ≤ 5	MlogP ≤ 5			
1	Succinate	116.07	4	0	−0.54	0	0.56	80.26
2	Acetate	59.04	2	0	−0.49	0	0.85	40.13
3	Baicalein	270.24	5	3	0.52	0	0.55	90.9
4	Baicalin	446.36	11	6	−1.63	2	0.11	187.12
5	Butyrate	87.1	2	0	0.49	0	0.85	40.13
6	3-Indolepropionic acid	189.21	2	2	1.4	0	0.85	53.09
7	Trimethylamine oxide	75.11	1	0	−1.66	0	0.55	29.43
8	Propionate	73.07	2	0	0.03	0	0.85	40.13
9	Equol	242.27	3	2	2.2	0	0.55	49.69
10	Phenylacetylglutamine	264.28	4	3	0.4	0	0.56	109.49
11	Indole	117.15	0	1	1.57	0	0.55	15.79
12	Oxindole	133.15	1	1	1.13	0	0.55	29.1
13	Indole-3-acrylic acid	187.19	2	2	1.32	0	0.85	53.09
14	3-Methylindole	131.17	0	1	1.89	0	0.55	15.79
15	Kynurenic acid	189.17	3	2	0.41	0	0.85	70.16
16	Urolithin A	228.2	4	2	1.68	0	0.55	70.67
17	Equol	242.27	3	2	2.2	0	0.55	49.69
18	Indoxyl sulfate	213.21	4	2	0.22	0	0.56	87.77
19	p-Cresol sulfate	188.2	4	1	1	0	0.85	71.98
20	10-Oxo-11-octadecenoic acid	296.44	3	1	3.59	0	0.85	54.37
21	Lithocholic acid	376.57	3	2	4.73	1	0.85	57.53
22	2,2′,4,4′,5-Pentabromodiphenyl ether	564.69	1	0	6.55	2	0.17	9.23
23	10-Keto-12Z-octadecenoic acid	296.44	3	1	3.59	0	0.85	54.37

**Table 2:** Toxicological properties of the metabolites from the gut microbiota.

NO.	Metabolites	hERG blockers	H-HT	Respiratory toxicity	Rat oral acute toxicity	Carcinogenicity	DILI	Skin sensitization	LD50
1	Succinate	−−−	+++	−−−	−−−	−−	−−−	+	3.117
2	Acetate	−−−	+++	−−−	−−−	−−	−−−	+	2.597
3	Baicalein	−−−	−−−	−	−−	−	+++	+++	5.607
4	Butyrate	−−−	++	−−−	−−	−	−−−	−	3.301
5	3-Indolepropionic acid	−−−	−−	−	++	−−	−−	−	4.475
6	Trimethylamine oxide	−−−	+	−−−	−−−	−−−	−−−	−−−	1.887
7	Propionate	−−−	++	−−−	−−−	−−	−−−	+	3.158
8	Equol	−−−	−−−	−−−	−−	++	−−−	+++	5.238
9	Phenylacetylglutamine	−−−	−−−	−−−	−−−	−−−	−	−−	3.038
10	Indole	−−−	−−	+++	++	−	−	+	5.328
11	Oxindole	−−−	−	−−	−−	+	+++	++	3.318
12	Indole-3-acrylic acid	−−−	+	+++	+++	−−	++	+++	4.725
13	3-Methylindole	−−−	−	++	++	−−	−	+	5.66
14	Kynurenic acid	−−−	−−	+++	−	−−−	+++	−−	4.285
15	Urolithin A	−−−	−−	−−	−−	+	+++	+++	5.129
16	Equol	−−−	−−−	−−−	−−	++	−−−	+++	5.238
17	Indoxyl sulfate	−−−	++	+++	+++	−	−−−	++	4.42
18	p-Cresol sulfate	−−−	+++	−	−−−	+	−−−	+	3.567
19	10-Oxo-11-octadecenoic acid	−−−	+	+++	−−−	−	−−−	+++	4.242
20	10-Keto-12Z-octadecenoic acid	−−−	−−−	−	−−−	−−	−−−	+	3.806

For the classification endpoints, the prediction probability values are transformed into six symbols: 0–0.1(−−−), 0.1–0.3(−−), 0.3–0.5(−), 0.5–0.7(+), 0.7–0.9(++), and 0.9–1.0(+++).

### 3.5 Molecular DOCKING validation

We selected the most promising metabolite, *Phenylacetylglutamine*, for molecular docking validation with CXCL8, IL6, and EGFR. The lower the free binding energy, the higher the binding activity and the more stable the binding conformation, indicating a greater likelihood of interaction. The binding energy between *Phenylacetylglutamine* and CXCL8 (PDB: 6WZM) was −6.6 kcal/mol (Figure 5B), with IL6 (PDB: 4O9H) it was −5.7 kcal/mol (Figure 5C), and with EGFR (PDB: 1XKK) it was −7.8 kcal/mol (Figure 5D). These results suggest a significant potential for interaction, highlighting the stability and efficacy of these molecular conformations.

### 3.6 Mendelian randomization study

We further conducted a Mendelian randomization analysis on gut microbiota metabolites, their upstream gut microbiota, and downstream related genes for causal inference. The genetic information for the gut microbiota metabolite *Phenylacetylglutamine* is identified as ebi-a-GCST90026248, comprising 291 samples and 6,873,547 SNPs. The upstream gut microbiota *Lachnospiraceae* (Kurilshikov et al., 2021) is cataloged as ebi-a-GCST90016940, with 14,306 samples and 5,729,268 SNPs. The downstream related gene CXCL8 is listed as prot-b-11, with 3,394 samples and 5,270,646 SNPs; EGFR as prot-a-909, with 3,301 samples and 10,534,735 SNPs; IL6 as prot-b-2, with 3,394 samples and 5,270,646 SNPs; and the genetic information for COPD is ebi-a-GCST90018807, including 468,475 samples and 24,180,654 SNPs. All data sets pertain to European populations.

**Figure 6:**
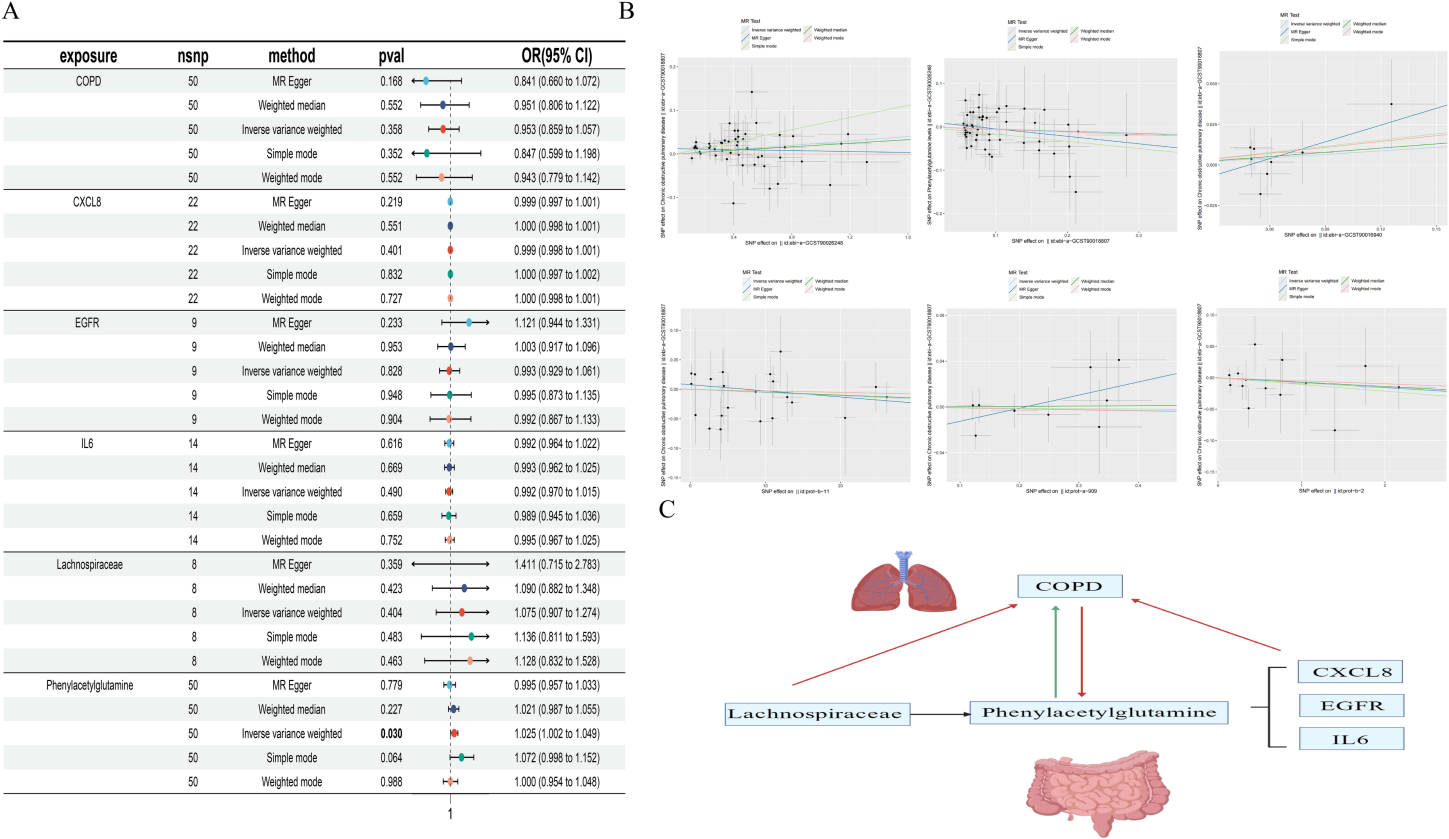
Forest plot of MR Analysis **(A)**; Scatter plot of MR Analysis **(B)**; Causal diagram of gut microbiota, gut microbiota metabolites and related genes with COPD (red is no causal relationship, green is causal relationship) **(C)**.

Through the selection of instrumental variables, we conducted an association analysis, removed linkage disequilibrium and weak instrumental variables, and discovered a causal relationship between the gut microbiome metabolite *Phenylacetylglutamine* and an increased risk of COPD (OR [95% CI], 1.025 [1.002–1.049], *p* = 0.03). Unfortunately, the reverse MR analysis did not show a causal relationship (OR [95% CI], 0.953 [0.859–1.057], *p* = 0.36); there is no causal relationship between the gut microbiome *Lachnospiraceae* and COPD (OR [95% CI], 1.074 [0.907–1.274], *p* = 0.40); and no causal relationships were found with downstream related genes CXCL8, EGFR, and IL6 in relation to COPD. Concurrently, tests for pleiotropy and heterogeneity were conducted, both exceeding 0.05, and the leave-one-out method validated the robustness of the MR results (Table 3; Figure 6).

**Table 3:** MR analysis.

Exposure	Method	Snp	Beta	Se	*p*-val	BWMR	Pleiotropy	Heterogeneity
Phenylacetylglutamine	IVW	50	0.030	0.031	0.030	0.025	0.058	0.745
Lachnospiraceae	IVW	8	0.072	0.087	0.404	0.431	0.448	0.734
CXCL8	IVW	22	−0.0005	0.0006	0.401	0.415	0.337	0.643
EGFR	IVW	9	−0.007	0.033	0.828	0.917	0.176	0.537
IL6	IVW	14	−0.008	0.012	0.490	0.492	0.977	0.876
COPD	IVW	50	−0.048	0.059	0.358	0.373	0.448	0.734

## 4 Discussion

In our investigation, network pharmacology studies revealed a comprehensive network analysis comprising 39 gut microbiota, 23 gut microbiota metabolites, 19 targets, and 20 signaling pathways associated with COPD. Further exploration into the drug-likeness and toxicology of gut microbiota metabolites identified *Phenylacetylglutamine* as a promising metabolite, substantiated by molecular docking validation. Causal inference regarding the relationship between the gut microbiota metabolite *Phenylacetylglutamine*, the upstream gut microbiota *Lachnospiraceae*, and downstream related genes CXCL8, EGFR, and IL6 with COPD revealed a positive causal relationship between *Phenylacetylglutamine* and COPD. However, reverse MR analysis did not support this causal relationship, and no direct causal links were found between the upstream gut microbiota, related targets, and COPD.

PPI networks and the gut microbiota-metabolite-target-signaling pathway network identified CXCL8, IL6, and EGFR as crucial targets closely associated with COPD. Dysbiosis of the gut microbiota is linked to systemic inflammation, with IL6 levels correlating with the abundance of various gut microbiota (Zhao et al., 2023). The anti-inflammatory effects of the gut microbiota may be mediated by the endocannabinoid system (Vijay et al., 2021), suggesting that modulation of the gut microbiota could reduce IL6 levels, thereby restoring immunity, reducing pulmonary inflammation (Deng et al., 2024), and alleviating asthma (Liu et al., 2023) and COPD (Wang J. et al., 2023). IL6 is upregulated in the lower airways of patients with mild to moderate COPD (Sulaiman et al., 2023), and IL-6TS could play a pathogenic role in chronic respiratory diseases (Winslow et al., 2021). CXCL8, a primary mediator of inflammatory responses, plays a role in systemic inflammation, and reducing CXCL8 levels could inhibit COPD inflammation (Castellucci et al., 2015). EGFR is capable of engaging in numerous cellular responses, serving as an integral component of the cytokine storm, and is implicated in the onset of severe acute respiratory distress syndrome. Decreasing EGFR levels may alleviate chronic bronchial inflammation (Wang C. C. et al., 2024), while cigarette smoke can activate EGFR (Muratani et al., 2023), thereby modulating the abnormal airway remodeling in COPD (Strickson et al., 2023). These targets may serve as potential therapeutic targets for treating COPD through gut microbiota and metabolites.

DO enrichment analysis of core targets indicated a significant association with respiratory diseases such as obstructive lung disease, chronic obstructive pulmonary disease, bronchial disease, and asthma, underscoring the regulatory potential of gut microbiota and metabolites on COPD through these targets. GO enrichment analysis revealed that gut microbiota metabolites and COPD-related targets are primarily associated with responses to xenobiotic stimuli, secondary metabolic processes, cellular responses to growth factor stimuli, responses to lipopolysaccharide, regulation of smooth muscle cell proliferation, responses to oxidative stress, and regulation of inflammatory responses, thereby modulating COPD. Oxidative stress exacerbates COPD (Dal Negro et al., 2017), which is related to chronic inflammation of the lung parenchyma and peripheral airways (Barnes, 2016), and can be mediated by regulating airway smooth muscle damage and endoplasmic reticulum stress (Chen et al., 2022). Inflammation and oxidative stress play key roles in the pathogenesis and progression of COPD (Wiegman et al., 2015). Thus, gut microbiota metabolites can alleviate COPD through multiple mechanisms, including oxidative stress, inflammatory responses, and smooth muscle modulation, revealing their functional role in treating COPD. KEGG enrichment analysis showed that gut microbiota and metabolites regulate COPD mechanisms through signaling pathways such as Lipid and atherosclerosis, IL-17 signaling pathway, NF-kappa B signaling pathway, PI3K-Akt signaling pathway, and JAK–STAT signaling pathway. Lipoprotein metabolism may be related to COPD (Santana et al., 2021), and lipid metabolism has a correlation with the pathogenesis of COPD (Kotlyarov and Kotlyarova, 2021), potentially offering a novel therapeutic approach (Sonett et al., 2018). The JAK–STAT signaling pathway plays a crucial role in activating cytokines in inflammatory responses in COPD (Yew-Booth et al., 2015), and the IL − 17 signaling pathway can coordinate lung immune defense in COPD (McAleer and Kolls, 2014). These pathways play significant roles in the development of COPD, warranting focused attention on the gut microbiota-metabolite-target-signaling pathway network.

In our investigation of 23 gut microbiome metabolites for their pharmacological similarity and toxicological profiles, we identified *Phenylacetylglutamine* as the most promising metabolite. Research suggests that *Phenylacetylglutamine* could serve as a prognostic marker for heart failure risk (Tang et al., 2024), is associated with various cardiovascular disease risks (Song et al., 2024), and correlates with multiple diseases (Krishnamoorthy et al., 2024). Studies on the relationship between COPD and *Phenylacetylglutamine* are sparse, yet some research indicates that *Phenylacetylglutamine* may act as a potential biomarker for lung cancer (Aredo et al., 2021), inhibiting the growth of lung tumors (Wang et al., 2012), and high levels of *Phenylacetylglutamine* may be linked to lung damage and Acute Respiratory Distress Syndrome (Xu et al., 2020). Our Mendelian randomization analysis of *Phenylacetylglutamine* and COPD revealed a positive causal relationship, suggesting that *Phenylacetylglutamine* could be a potential biomarker for treating COPD. *Lachnospiraceae* is somewhat associated with a healthy lifestyle (Zhang et al., 2015), participates in the progression of various diseases (Gomes et al., 2018; Zuo et al., 2019), is related to the release of inflammatory cytokines (Schirmer et al., 2016), and correlates with the disease characteristics of COPD (Budden et al., 2024). In mice with COPD induced by CS, there is an increased relative abundance of *Lachnospiraceae* (Lai et al., 2022b). We discovered that the upstream gut microbiota *Lachnospiraceae*, involved in various carbohydrate metabolisms, could provide energy for the body. *Lachnospiraceae* can modulate immunity to protect against influenza’s pulmonary assault (McCumber et al., 2024), is associated with lung inflammation in COPD (Budden et al., 2024), participates in cytokine regulation altering COPD’s lung inflammation (Wu et al., 2023), and is related to various respiratory diseases such as lung cancer (Zhang et al., 2024), pulmonary fibrosis (Sun et al., 2024b), cough (Liu Y. et al., 2024), and asthma (Wang et al., 2024a). It can suppress inflammation and apoptosis, mitigating the progression of emphysema (Jang et al., 2020), potentially serving as a novel paradigm for treating COPD. However, it is regrettable that Mendelian randomization showed no causal relationship between *Lachnospiraceae* and COPD.

In the future, we will validate the gut microbiome metabolite Phenylacetylglutamine in both COPD patients and healthy groups, referencing related literature (Nemet et al., 2020). Further, through *in vivo* and *in vitro* experiments, we aim to elucidate the potential mechanisms of *Phenylacetylglutamine* in COPD patients. By integrating extracellular vesicles (Lai et al., 2022a) and exosomes (Li et al., 2018), the mRNA within these structures may predict potential biomarkers for various diseases (Li et al., 2019; Li Y. et al., 2021). This approach will also be used to predict potential biomarkers for COPD, further suggesting that *Phenylacetylglutamine* could represent a novel therapeutic avenue for COPD treatment.

## 5 Conclusion

This study, employing network pharmacology and Mendelian randomization, delves into the metabolites of the gut microbiome in the treatment of COPD, elucidating the intricate connections between the gut microbiome, its metabolites, and COPD. It identifies *Phenylacetylglutamine*, *Lachnospiraceae*, core targets, and signaling pathways as novel avenues for COPD treatment. Further exploration of their causal relationships with COPD revealed that, aside from a causal relationship between *Phenylacetylglutamine* and COPD, no other causal relationships were found. These discoveries aid in the screening of individuals at high risk for COPD, offering insights into early prevention and treatment strategies for the disease.

## Data Availability

The original contributions presented in the study are included in the article/Supplementary material, further inquiries can be directed to the corresponding author.

## Footnotes

^1^ http://bio-annotation.cn/gutmgene/

^2^ https://pubchem.ncbi.nlm.nih.gov/

^3^ https://sea.bkslab.org/

^4^ http://www.swisstargetprediction.ch/

^5^ https://www.genecards.org/

^6^ https://www.omim.org/

^7^ https://go.drugbank.com/

^8^ https://string-db.org/

^9^ http://www.swissadme.ch/

^10^ https://admetlab3.scbdd.com/

^11^ https://gwas.mrcieu.ac.uk/
