# Supplementary material for: Potential of gut microbiota metabolites in treating COPD: network pharmacology and Mendelian randomization approaches
Source: Front Microbiol. 2024 Nov 25;15:1416651. doi: 10.3389/fmicb.2024.1416651 (PMC11625750; doi:10.3389/fmicb.2024.1416651)
Supplement: Supplementary file 1 [file Data_Sheet_1.PDF]

STROBE-MR checklist of recommended items to address in reports of Mendelian randomization studies[1 2]

Item No.	Section		Checklist item	Relevant text from manuscript
1	**TITLE and ABSTRACT**		Indicate Mendelian randomization (MR) as the study's design in the title and/or the abstract if that is a main purpose of the study	Potential of Gut Microbiota Metabolites in Treating COPD: Network Pharmacology and Mendelian Randomization Approaches
	**INTRODUCTION**			
2	**Background**		Explain the scientific background and rationale for the reported study. What is the exposure? Is a potential causal relationship between exposure and outcome plausible? Justify why MR is a helpful method to address the study question	Studies have shown that the gut microbiome plays a role in the pathogenesis of COPD and could potentially be targeted for therapeutic interventions.
3	**Objectives**		State specific objectives clearly, including pre-specified causal hypotheses (if any). State that MR is a method that, under specific assumptions, intends to estimate causal effects	The gut microbiota and its metabolites exert a significant influence on COPD, yet the underlying mechanisms remain elusive. We aim to holistically evaluate the role and mechanisms of the gut microbiota and its metabolites in COPD through network pharmacology and Mendelian randomization approaches.
	**METHODS**			
4	**Study design and data sources**		Present key elements of the study design early in the article. Consider including a table listing sources of data for all phases of the study. For each data source contributing to the analysis, describe the following:	
		a)	Setting: Describe the study design and the underlying population, if possible. Describe the setting, locations, and relevant dates, including periods of recruitment, exposure, follow-up, and data collection, when available.	We further conducted a Mendelian randomization analysis on gut microbiota metabolites, their upstream gut microbiota, and downstream related genes for causal inference. The genetic information for the gut microbiota metabolite Phenylacetylglutamine is identified as ebi-a-GCST90026248, comprising 291 samples and 6,873,547 SNPs. The upstream gut microbiota Lachnospiraceae is cataloged as ebi-a-GCST90016940, with 14,306 samples and 5,729,268 SNPs. The downstream related gene CXCL8 is listed as prot-b-11, with 3,394 samples and 5,270,646 SNPs; EGFR as prot-a-909, with 3,301 samples and 10,534,735 SNPs; IL6 as prot-b-2, with 3,394 samples and 5,270,646 SNPs; and the genetic information for COPD is ebi-a-GCST90018807, including 468,475 samples and 24,180,654 SNPs. All data sets pertain to

				European populations.
		b)	Participants: Give the eligibility criteria, and the sources and methods of selection of participants. Report the sample size, and whether any power or sample size calculations were carried out prior to the main analysis	The genetic information for the gut microbiota metabolite Phenylacetylglutamine is identified as ebi-a-GCST90026248, comprising 291 samples and 6,873,547 SNPs. The upstream gut microbiota Lachnospiraceae is cataloged as ebi-a-GCST90016940, with 14,306 samples and 5,729,268 SNPs. The downstream related gene CXCL8 is listed as prot-b-11, with 3,394 samples and 5,270,646 SNPs; EGFR as prot-a-909, with 3,301 samples and 10,534,735 SNPs; IL6 as prot-b-2, with 3,394 samples and 5,270,646 SNPs; and the genetic information for COPD is ebi-a-GCST90018807, including 468,475 samples and 24,180,654 SNPs. All data sets pertain to European populations.
		c)	Describe measurement, quality control and selection of genetic variants	Firstly, due to the restricted pool of SNPs accessible for MR analysis, a significance threshold of P value was less than 5×10^{-6} was established for the detection of SNPs that exhibit strong associations with the investigated exposures. Moreover, to eliminate any presence of linkage disequilibrium, an r2 threshold of 0.001 and a clump window size of 10,000 kb were implemented.
		d)	For each exposure, outcome, and other relevant variables, describe methods of assessment and diagnostic criteria for diseases	Not Applicable
		e)	Provide details of ethics committee approval and participant informed consent, if relevant	Not Applicable
5	**Assumptions**		Explicitly state the three core IV assumptions for the main analysis (relevance, independence and exclusion restriction) as well assumptions for any additional or sensitivity analysis	In the MR analysis, SNPs were considered as IVs. These IVs needed to satisfy three core assumptions: the hypothesis of correlation, the hypothesis of exclusivity, and the assumption of Independence. The first assumption establishes a robust link between SNPs and the variable of exposure. Secondly, the selected SNPs were ensured to have no association with any confounding factors that could influence the relationship between exposure and outcome. Lastly, the SNPs were confirmed to only impact the outcome through exposure factors.

6	**Statistical methods: main analysis**		Describe statistical methods and statistics used	
		a)	Describe how quantitative variables were handled in the analyses (i.e., scale, units, model)	This research does not involve any transformations of quantitative variables.
		b)	Describe how genetic variants were handled in the analyses and, if applicable, how their weights were selected	The genetic information pertaining to the gut microbiome metabolites, their upstream microbiota, and downstream genes in relation to COPD is sourced from the GWAS database . To evaluate the causality, we employ five methods: Inverse Variance Weighted (IVW), MR-Egger, Weighted Median, Simple Mode, and Weighted Mode methods, with IVW serving as the primary method. A p-value of less than 0.05 indicates a causal relationship while the other four methods serve as supplementary approaches.
		c)	Describe the MR estimator (e.g. two-stage least squares, Wald ratio) and related statistics. Detail the included covariates and, in case of two-sample MR, whether the same covariate set was used for adjustment in the two samples	Genetic associations with all exposures were taken from a large meta-analysis of GWAS, we obtained SNP-specific Wald estimates and then used inverse variance weighting (IVW) with multiplicative random effects, MR-Egger, and weighted median (WM). The IVW method is a classical method for MR analysis, where the weighted average is calculated by taking the reciprocal of the variance of each IV as the weight, ensuring the effectiveness of all IVs. MR-Egger utilizes a weighted linear regression analysis, providing robust estimates that are independent of the validity of instrumental variables. Nevertheless, it is crucial to acknowledge that these estimates may have lower statistical precision and can be influenced by outlier genetic variation. On the other hand, The problem of estimation accuracy variability is tackled by the WM approach. In a manner reminiscent of the IVW approach, the WM method assigns inverse weights that are contingent upon the variance of individual genetic variants, demonstrating reliability even when causal effects are violated.
		d)	Explain how missing data were addressed	In this MR analysis, the issue of missing data was not involved.
		e)	If applicable, indicate how multiple testing was addressed	In this MR analysis, multiple exposures or multiple outcomes were not involved, so multiple testing was not performed.

7	**Assessment of assumptions**	Describe any methods or prior knowledge used to assess the assumptions or justify their validity	To assess the robustness of our results, leave-one-out sensitivity analysis is conducted, further complemented by tests for pleiotropy and heterogeneity, with a p-value greater than 0.05 indicating the absence of both pleiotropy and heterogeneity.
8	**Sensitivity analyses and additional analyses**	Describe any sensitivity analyses or additional analyses performed (e.g. comparison of effect estimates from different approaches, independent replication, bias analytic techniques, validation of instruments, simulations)	To assess the robustness of our results, leave-one-out sensitivity analysis is conducted, further complemented by tests for pleiotropy and heterogeneity, with a p-value greater than 0.05 indicating the absence of both pleiotropy and heterogeneity. All analyses are performed using the R programming language (version 4.3.2).
9	**Software and pre-registration**		
	a)	Name statistical software and package(s), including version and settings used	All analyses were conducted using R version 4.3.2, with the software packages 'Two-SampleMR'. To visualize the MR analysis, forest plots, scatter plots, and leave-one-out plots were generated using the data analysis function of the Rstudio platform.
	b)	State whether the study protocol and details were pre-registered (as well as when and where)	This study was not pre-registered with the study protocol and details.
	**RESULTS**		
10	**Descriptive data**		
	a)	Report the numbers of individuals at each stage of included studies and reasons for exclusion. Consider use of a flow diagram	The genetic information for the gut microbiota metabolite Phenylacetylglutamine is identified as ebi-a-GCST90026248, comprising 291 samples and 6,873,547 SNPs. The upstream gut microbiota Lachnospiraceae is cataloged as ebi-a-GCST90016940, with 14,306 samples and 5,729,268 SNPs. The downstream related gene CXCL8 is listed as prot-b-11, with 3,394 samples and 5,270,646 SNPs; EGFR as prot-a-909, with 3,301 samples and 10,534,735 SNPs; IL6 as prot-b-2, with 3,394 samples and 5,270,646 SNPs; and the genetic information for COPD is ebi-a-GCST90018807, including 468,475 samples and 24,180,654 SNPs.
	b)	Report summary statistics for phenotypic exposure(s), outcome(s), and other relevant variables (e.g. means, SDs, proportions)	Summary data on exposure and outcomes are shown in Table 3.
	c)	If the data sources include meta-analyses of previous studies, provide the assessments of heterogeneity across these studies	Summary data on exposure and outcomes are shown in Table 3.

		d)	For two-sample MR:<br>i. Provide justification of the similarity of the genetic variant-exposure associations between the exposure and outcome samples<br>ii. Provide information on the number of individuals who overlap between the exposure and outcome studies	The data presented in this study were derived exclusively from European population samples. These samples were obtained from independent GWAS databases, ensuring minimal overlap and bias, detailed data on the number of individuals in the exposure and outcome samples are provided in Table 3.
11	**Main results**			
		a)	Report the associations between genetic variant and exposure, and between genetic variant and outcome, preferably on an interpretable scale	Through meticulous selection of tools for associative analysis, elimination of linkage disequilibrium, and weak instrument variables, we discovered a causal relationship between the gut microbiota metabolite Phenylacetylglutamine and an increased risk of COPD (OR [95% CI], 1.025 [1.002-1.048], P = 0.03). Regrettably, reverse MR analysis did not reveal a causal relationship (OR [95% CI], 0.953 [0.859-1.057], P = 0.36); no causal relationship was found between the gut microbiota Lachnospiraceae and COPD (OR [95% CI], 1.074 [0.907-1.273], P = 0.40); nor was a causal relationship identified with the downstream related genes CXCL8, EGFR, and IL6 in relation to COPD. Tests for pleiotropy and heterogeneity were conducted, both yielding results >0.05, and leave-one-out sensitivity analysis confirmed the robustness of the MR findings.
		b)	Report MR estimates of the relationship between exposure and outcome, and the measures of uncertainty from the MR analysis, on an interpretable scale, such as odds ratio or relative risk per SD difference	Mengelian randomization estimation reports are detailed in Table 3.
		c)	If relevant, consider translating estimates of relative risk into absolute risk for a meaningful time period	Not Applicable
		d)	Consider plots to visualize results (e.g. forest plot, scatterplot of associations between genetic variants and outcome versus between genetic variants and exposure)	The results are visualized in Figure 6.
12	**Assessment of assumptions**			
		a)	Report the assessment of the validity of the assumptions	First, we have selected SNPs derived from network pharmacology of intestinal flora, intestinal flora metabolites, and targets as instrumental variables. Second, we ensure that the selected SNPs are not associated with any confounding factors that could potentially influence the relationship between exposure and outcome. Finally, these SNPs have been confirmed

				to affect the outcome only through the exposure factor.
		b)	Report any additional statistics (e.g., assessments of heterogeneity across genetic variants, such as I^2, Q statistic or E-value)	See Supplementary Table 3 for details.
13	**Sensitivity analyses and additional analyses**			
		a)	Report any sensitivity analyses to assess the robustness of the main results to violations of the assumptions	See Supplementary Table 3 for details.
		b)	Report results from other sensitivity analyses or additional analyses	See Supplementary Table 3 for details.
		c)	Report any assessment of direction of causal relationship (e.g., bidirectional MR)	See Supplementary Table 3 for details.
		d)	When relevant, report and compare with estimates from non-MR analyses	This study does not involve non-MR studies.
		e)	Consider additional plots to visualize results (e.g., leave-one-out analyses)	See Supplementary Figure 6 for details.
	**DISCUSSION**			
14	**Key results**		Summarize key results with reference to study objectives	We discovered a causal relationship between the gut microbiota metabolite Phenylacetylglutamine and an increased risk of COPD. Regrettably, reverse MR analysis did not reveal a causal relationship ; no causal relationship was found between the gut microbiota Lachnospiraceae and COPD ; nor was a causal relationship identified with the downstream related genes CXCL8, EGFR, and IL6 in relation to COPD.
15	**Limitations**		Discuss limitations of the study, taking into account the validity of the IV assumptions, other sources of potential bias, and imprecision. Discuss both direction and magnitude of any potential bias and any efforts to address them	Firstly, the databases used in the study only included individuals of European ancestry. To obtain stronger evidence, it is necessary to expand the databases to include other ethnic groups such as those from Asia and Africa. Secondly, the threshold of P value was less than 5×10^{-8} is generally considered to indicate genome-wide significance when screening for IVs. However, in this study, the threshold of P value was set less than 5×10^{-6} in order to obtain a sufficient number of SNPs. It is important to interpret the study results with caution, as this difference in threshold may have some impact on the findings. Third, the MR analysis method is a theoretical causal analysis method that requires further validation through animal experiments to establish the causal relationship.

16	**Interpretation**		
		a) Meaning: Give a cautious overall interpretation of results in the context of their limitations and in comparison with other studies	Further exploration of their causal relationships with COPD revealed that, aside from a causal relationship between Phenylacetylglutamine and COPD, no other causal relationships were found. These discoveries aid in the screening of individuals at high risk for COPD, offering insights into early prevention and treatment strategies for the disease.
		b) Mechanism: Discuss underlying biological mechanisms that could drive a potential causal relationship between the investigated exposure and the outcome, and whether the gene-environment equivalence assumption is reasonable. Use causal language carefully, clarifying that IV estimates may provide causal effects only under certain assumptions	In this study, the methodology of network pharmacology is employed to explore the relationships between the gut microbiome, its metabolites, targets, signaling pathways, and COPD from a holistic perspective. The aim is to identify the core gut microbiome, its metabolites,targets, and key signaling pathways that influence COPD. Furthermore, by utilizing the Mendelian randomization approach, the study delves into the causal relationships between the core gut microbiome, its metabolites, and targets with COPD, thereby dissecting the causality between these elements and COPD from an overarching standpoint.
		c) Clinical relevance: Discuss whether the results have clinical or public policy relevance, and to what extent they inform effect sizes of possible interventions	This study illuminates the intricate associations between the gut microbiota, its metabolites, and COPD. Phenylacetylglutamine may represent a novel avenue for COPD treatment. These findings could aid in identifying individuals at high risk for COPD, offering insights into early prevention and treatment strategies.
17	**Generalizability**	Discuss the generalizability of the study results (a) to other populations, (b) across other exposure periods/timings, and (c) across other levels of exposure	The selected gut microbiome metabolites, along with their upstream gut microbiota and downstream relevant genes, will be subjected to Mendelian randomization to ascertain their causal relationship with COPD. However, it did not investigate the effects of varying exposure periods or levels. Furthermore, the study was limited to a European population, raising questions about its generalizability to other populations.
	**OTHER INFORMATION**		
18	**Funding**	Describe sources of funding and the role of funders in the present study and, if applicable, sources of funding for the databases and original study or studies on which the present study is based	This study was funded by the Natural Science Foundation of Jilin Province (YDZJ202201ZYTS236, 20180101115JC and 20230203189SF), Administration of Traditional Chinese Medicine (2022ZYLCYJ04-1 and 202209), and Jilin Provincial Administration of

			Traditional Chinese Medicine (2022219).
19	**Data and data sharing**	Provide the data used to perform all analyses or report where and how the data can be accessed, and reference these sources in the article. Provide the statistical code needed to reproduce the results in the article, or report whether the code is publicly accessible and if so, where	The original contributions presented in the study are included in the article, further inquiries can be directed to the corresponding authors.
20	**Conflicts of Interest**	All authors should declare all potential conflicts of interest	The authors declare that the research was conducted in the absence of any commercial or financial relationships that could be construed as potential conflicts of interest.

This checklist is copyrighted by the Equator Network under the Creative Commons Attribution 3.0 Unported (CC BY 3.0) license.

1. Skrivankova VW, Richmond RC, Woolf BAR, Yarmolinsky J, Davies NM, Swanson SA, et al. Strengthening the Reporting of Observational Studies in Epidemiology using Mendelian Randomization (STROBE-MR) Statement. JAMA. 2021;under review.

2. Skrivankova VW, Richmond RC, Woolf BAR, Davies NM, Swanson SA, VanderWeele TJ, et al. Strengthening the Reporting of Observational Studies in Epidemiology using Mendelian Randomisation (STROBE-MR): Explanation and Elaboration. BMJ. 2021;375:n2233.
